# A comparison of medium-term heat acclimation by post-exercise hot water immersion or exercise in the heat: adaptations, overreaching, and thyroid hormones

**DOI:** 10.1152/ajpregu.00315.2021

**Published:** 2022-09-12

**Authors:** Robert D. McIntyre, Michael J. Zurawlew, Jessica A. Mee, Neil P. Walsh, Samuel J. Oliver

**Affiliations:** ^1^School of Human and Behavioural Sciences, Bangor University, Bangor, United Kingdom; ^2^School of Sport and Exercise Sciences, Liverpool John Moores University, Liverpool, United Kingdom; ^3^School of Sport and Exercise Science, University of Worcester, Worcester, United Kingdom

**Keywords:** core temperature, hot bath, thermoregulation, thyroxine, triiodothyronine

## Abstract

This research compared thermal and perceptual adaptations, endurance capacity, and overreaching markers in men after 3, 6, and 12 days of post-exercise hot water immersion (HWI) or exercise heat acclimation (EHA) with a temperate exercise control (CON), and examined thyroid hormones as a mechanism for the reduction in resting and exercising core temperature (*T*_re_) after HWI. HWI involved a treadmill run at 65% V̇o_2peak_ at 19°C followed by a 40°C bath. EHA and CON involved a work-matched treadmill run at 65% V̇o_2peak_ at 33°C or 19°C, respectively. Compared with CON, resting mean body temperature (*T*_b_), resting and end-exercise *T*_re_, *T*_re_ at sweating onset, thermal sensation, and perceived exertion were lower and whole-body sweat rate (WBSR) was higher after 12 days of HWI (all *P* ≤ 0.049, resting *T*_b_: CON −0.11 ± 0.15°C, HWI −0.41 ± 0.15°C). Moreover, resting *T*_b_ and *T*_re_ at sweating onset were lower after HWI than EHA (*P* ≤ 0.015, resting *T*_b_: EHA −0.14 ± 0.14°C). No differences were identified between EHA and CON (*P* ≥ 0.157) except WBSR that was greater after EHA (*P* = 0.013). No differences were observed between interventions for endurance capacity or overreaching markers (mood, sleep, Stroop, *P* ≥ 0.190). Thermal adaptations observed after HWI were not related to changes in thyroid hormone concentrations (*P* ≥ 0.086). In conclusion, 12 days of post-exercise hot water immersion conferred more complete heat acclimation than exercise heat acclimation without increasing overreaching risk, and changes in thyroid hormones are not related to thermal adaptations after post-exercise hot water immersion.

## Graphical abstract

**Figure:**
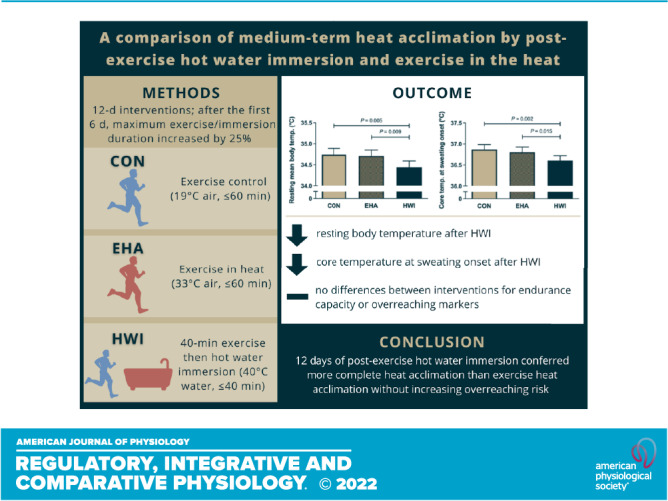

## INTRODUCTION

It is well established that exercise in hot and hot-humid environments is detrimental to endurance capacity (1, 2) and may expose individuals to the risk of exertional heat illness (3). To reduce these deleterious effects of heat stress, athletes, military personnel, and occupational workers should prepare by completing a period of heat acclimation (4, 5).

Previous research in both recreationally active (6) and endurance-trained individuals (7) demonstrates that taking a hot bath for up to 40 min immediately after submaximal exercise in temperate conditions on six consecutive days reduces resting core body temperature. This reduction in resting core body temperature leads to a subsequent reduction in core body temperature during exercise-heat stress, a hallmark heat acclimation adaptation. post-exercise hot water immersion (HWI) also presents a more practical heat acclimation strategy than conventional exercise heat acclimation (EHA), as it eliminates the requirement for access to an environmental chamber and can be more easily incorporated into normal training and an athlete’s taper (8). Moreover, McIntyre et al. (9) recently demonstrated that despite a similar endogenous thermal stimulus for adaptation, 6 days of HWI elicited larger thermal adaptations than EHA. Although the 6-day HWI intervention presents an effective, practical, and time-efficient short-term (< 7 days) heat acclimation strategy, previous literature suggests that medium- (7–14 days) (10, 11) and long-term (>14-day) (10) interventions provide a more complete state of heat acclimation. It is yet to be determined whether extending the 6-day HWI intervention provides additional thermal benefits. In addition, the true benefit of medium-term conventional exercise-based heat acclimation strategies beyond exercising in temperate conditions is unknown due to the lack of work-matched interventions within the literature and hence further research is warranted. In contrast to the beneficial adaptations of medium-term heat acclimation, the physical demands of prolonged interventions can disrupt training and may trigger overreaching, which has detrimental effects on exercise performance and mood (4, 12). The effects of medium-term heat acclimation on overreaching are currently unknown and, given the applied implications, warrant investigation.

The reduction in thermal strain after HWI heat acclimation can be largely attributed to a reduction in resting core temperature (6, 7, 9, 13, 14). The underlying mechanism for this reduction in resting core temperature is currently unknown but may involve a reduction in metabolic heat production via reduced circulating thyroid hormone concentrations (15), a decrease in the thermoregulatory balance point (16, 17), or hypothalamic neural network remodeling (18, 19). The release of thyroid-stimulating hormone by the anterior pituitary gland stimulates the release of two protein-iodine-bound hormones: triiodothyronine (T3) and thyroxine (T4). When unbound, free thyroid hormones are metabolically active and stimulate glucose uptake, gluconeogenesis, lipolysis, and thermogenesis (20). Reductions in thyroid hormones have been demonstrated after 3 weeks of heat exposure in rats (21, 22) and previous research also shows rats with lower circulating thyroid hormones have a lower core temperature at rest and during heat stress (23, 24). However, no study to date in humans has investigated the effect of heat acclimation on thyroid hormone concentrations or thyroid hormone influences on heat acclimation thermal adaptations. Specifically, it is unknown whether reductions in thyroid hormones are responsible for the pronounced reduction in resting core temperature observed after HWI heat acclimation (6, 7, 9).

This research is presented in two parts. *Part 1* compared heat acclimation thermal and endurance capacity adaptations, overreaching markers, and changes in plasma thyroid hormone concentrations after 3, 6, and 12 days of HWI and EHA with a work-matched temperate exercise control (CON) in 21 active males. Given larger thermal adaptations were observed after short-term HWI than short-term EHA (9), we hypothesized that extending the HWI intervention to 12 days would augment thermal adaptations and that these would confer more complete heat acclimation than EHA. In addition, we expected that compared with CON the high physical demands of daily exercise and heat stress during HWI and EHA would lead to increased markers of overreaching (i.e., low mood and physical/cognitive performance decrements). *Part 2* examined, in a larger cohort of 48 active males, the effect of 6 days of HWI in comparison with CON on plasma thyroid hormone concentrations, and additionally examined the relationship of thyroid hormone changes with hallmark heat acclimation adaptations. We hypothesized that 6 days of HWI would elicit reductions in plasma thyroid hormone concentrations and that these reductions would be associated with heat acclimation thermal adaptations, in particular a reduction in resting and exercising core temperature.

## METHODS

### Experimental Approaches

**Figure 1.**
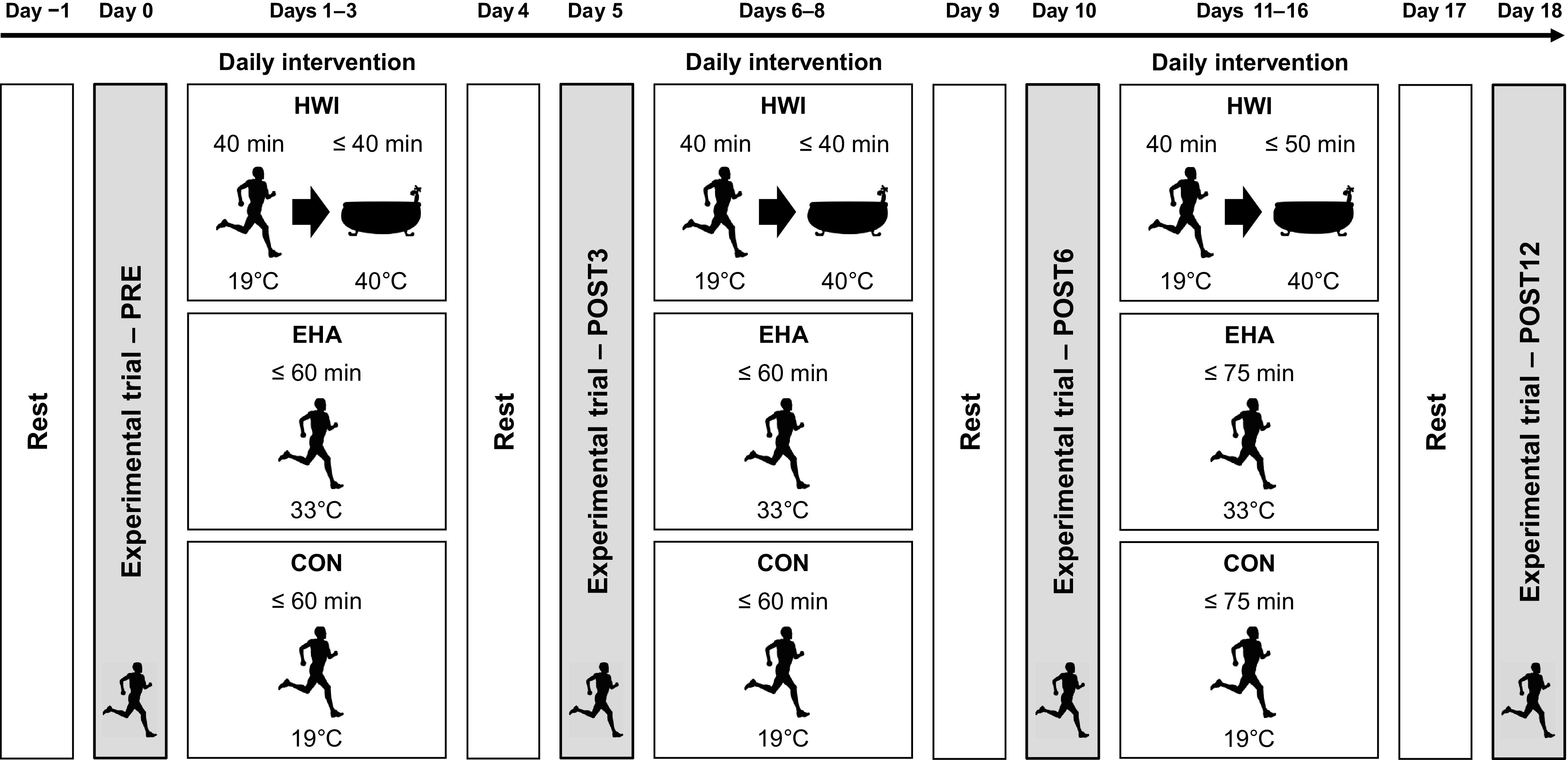
Schematic of the study design (*part 1*). CON; temperate exercise control; EHA, exercise heat acclimation; HWI, post-exercise hot water immersion.

In *part 1*, a mixed-methods (between and within) repeated-measures design was used to assess the effect of 12 days of HWI, EHA, and CON on thermal and perceptual adaptations, overreaching markers, and plasma thyroid hormone concentrations in 21 recreationally active males. This is a subset of participants of a larger cohort that completed six intervention days (9). Participants in *part 1* completed experimental trials before (PRE) and after 3 (POST3), 6 (POST6), and 12 days (POST12) of their assigned intervention (Fig. 1). To enable work-matching with EHA, CON participants completed the same external work ≥1 day after EHA participants. In *part 2*, data from four previously published heat acclimation studies from our laboratory (6, 7, 9, 14) were amalgamated in a between-groups design to assess the effect of 6 days of HWI and CON on thermal adaptations, thyroid hormone concentrations, and the relationship between plasma thyroid hormones and thermal adaptations. Thyroid hormones were not previously investigated in these studies. Amalgamating the four studies enabled the relationship between thyroid hormones and thermal adaptations to be examined more robustly in a larger sample. Testing was halted during summer months (June–August) to reduce the potential effect of seasonal heat acclimatization. All studies received ethical approval (829/MoDREC/17, PO5-17/18, S/PhD10-15/16, PhD19-13/14), were conducted in accordance with the Declaration of Helsinki (2013) but were not registered in a database.

### Participant Recruitment and Randomization

**Figure 2.**
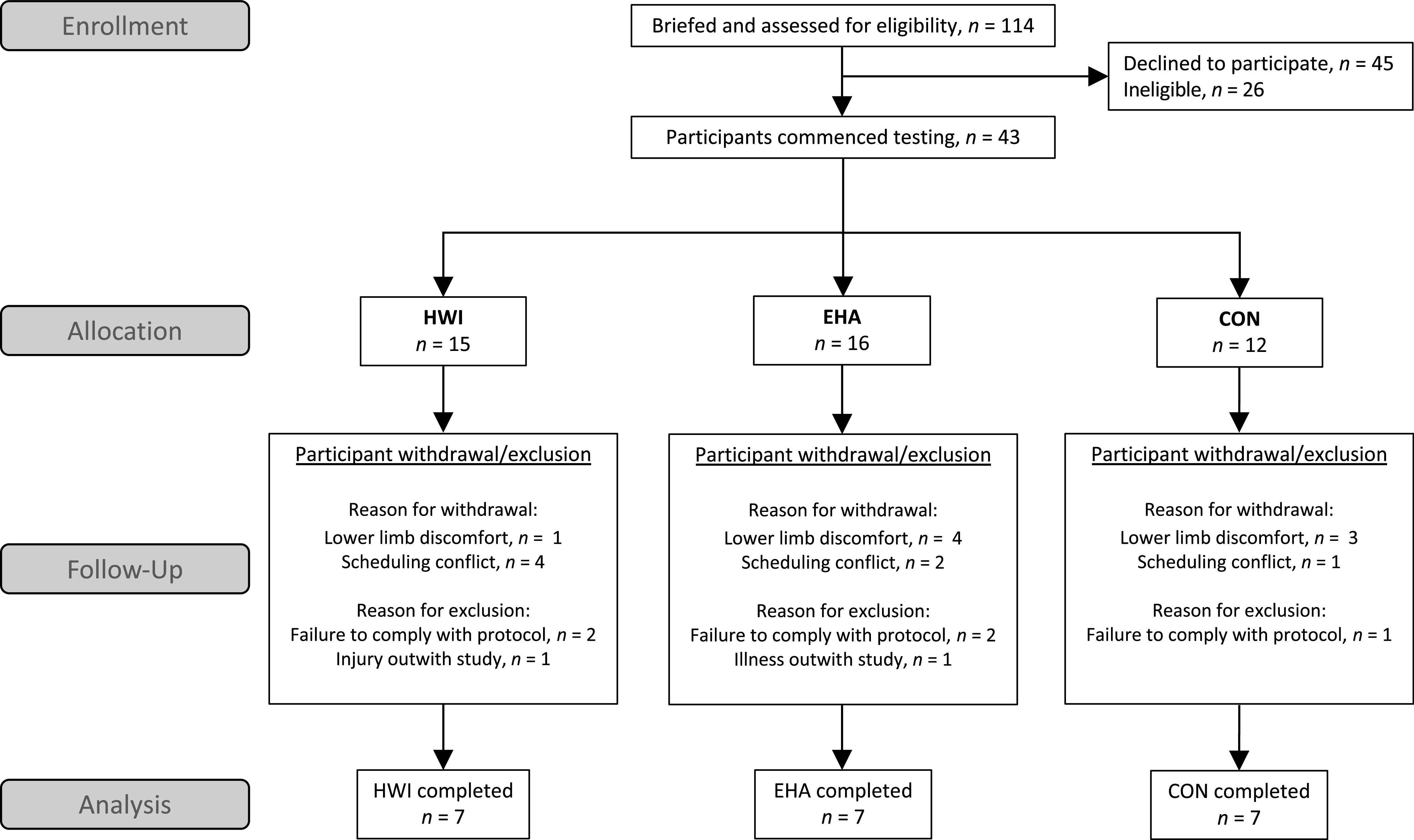
Flow diagram indicating the numbers of participants assessed for eligibility, commenced testing, and withdrew, were excluded, or completed the study protocol (*part 1*). CON, temperate exercise control; EHA, exercise heat acclimation; HWI, post-exercise hot water immersion.

*Part 1* participant flow and attrition before protocol completion and biochemical and statistical analyses are summarized in Fig. 2. Participants were matched for V̇o_2peak_ in groups of three and randomly assigned to either HWI, EHA, or CON (randomizer.org). Participants were excluded from the final analysis if they failed to complete the 12-day study protocol. The participant characteristics of the 21 male participants included in the final analysis are summarized in Table 1. A sample size of 21 (7 participants per group) was estimated (G*Power 3.1.9) (25) as adequate to detect a significant difference in the change in end-exercise rectal core temperature (*T*_re_) between heat acclimation and temperate exercise control interventions using a mixed-model analysis of covariance (ANCOVA), standard α (0.05), and power (0.80), and a Cohen’s *F* effect size of 0.88. This effect size was calculated from the average reduction in end-exercise *T*_re_ change after HWI (−0.36°C) (6) and exercise heat acclimation [−0.44°C (26) and −0.49°C (27)] compared with exercise in temperate conditions (0.00°C) (6) and a pooled SD of 0.21°C (control group) (6). *Part 2* participants were 48 active males (age, 22 ± 3 yr; height, 178 ± 6 cm; body mass, 72 ± 7 kg; V̇o_2peak_, 58 ± 8 mL·kg^−1^·min^−1^). Data of 14 participants (HWI, *n* = 7; CON, *n* = 7) were included in both *parts 1* and *2*. All participants in *parts 1* and *2* provided written informed consent and were healthy, nonsmokers, free from any known cardiovascular or metabolic diseases, were not taking any medication and had not been regularly (more than once a week) exposed to the heat (including sauna and hot bath use) in the 6 weeks before commencing testing.

**Table 1.** Part 1 participant characteristics of post-exercise hot water immersion, exercise heat acclimation, and temperate exercise control interventions

	HWI	EHA	CON
Age, yr	22 ± 3	21 ± 2	22 ± 2
Height, cm	176 ± 4	183 ± 5	177 ± 6
Body mass, kg	70 ± 6	75 ± 6	70 ± 7
V̇o_2peak_, mL·kg^−1^·min^−1^	53 ± 7	54 ± 3	53 ± 4
Running economy, kcal·kg^−1^·min^−1^	3.3 ± 0.1	3.6 ± 0.4	3.5 ± 0.3

Data are represented as means ± SD; *n* = 7 participants in each group. Analyzed by one-way ANOVA. CON, temperate exercise control; EHA, exercise heat acclimation; HWI, hot water immersion.

### Preliminary Measurements and Familiarization

Participants completed a fitness assessment within a week before their first experimental trial (PRE; Fig. 1). V̇o_2peak_ was assessed using a continuous maximal incremental exercise test performed on a motorized treadmill (HP Cosmos Mercury 4.0, Nussdorf-Traunstein, Germany) in a temperate laboratory (19°C, 45% relative humidity) to volitional exhaustion. V̇o_2peak_ was determined as the highest oxygen uptake attained over a 30-s period. The average values of breath-by-breath V̇o_2_ and V̇co_2_ during the final minute of each submaximal stage were used to calculate running economy, expressed as kilocalories per kilogram per minute (28). A running speed that elicited 65% V̇o_2peak_ in temperate conditions was subsequently determined by the interpolation of the running speed-V̇o_2_ relationship and confirmed via Douglas bag method. All participants ran at a speed below their anaerobic threshold as determined by the onset of blood lactate accumulation (29). Participants were then familiarized with the treadmill running speed, Stroop test, venepuncture, and Profile of Mood States (POMS) questionnaire.

### Experimental Trials

Twenty-four hours before the first experimental trial, participants were instructed to refrain from exercise, alcohol, diuretics, and caffeine and to complete a diet diary. Twenty-four hours before all subsequent experimental trials, participants were instructed to replicate this food and fluid intake. To ensure a similar circadian pattern, participants were instructed to sleep between 2200 and 0700 before experimental trials with their sleep duration and efficiency assessed by an Actigraph (Actigraph GT3X Version 4.4.0, Actigraph, Pensacola, FL). Sleep duration and efficiency were also assessed as overreaching markers (30).

On the day of the experimental trials, participants arrived at the laboratory at 0730 and were provided with a standardized breakfast (2,091 kJ, 71 g carbohydrate, 18 g fat, 17 g protein) and a bolus of water (7 mL·kg^−1^ of nude body mass). At 0800, dressed in a t-shirt, shorts, socks, and trainers, participants rested for 20 min in temperate conditions (19°C, 45% relative humidity). After the seated rest, participants completed the abbreviated POMS questionnaire (31) to determine total mood disturbance and energy index (vigor-fatigue) as markers of overreaching (30). A venous blood sample was then taken without stasis for the determination of plasma volume and plasma concentrations of free T3, free T4, total T3, and total T4. A urine sample was then analyzed to confirm urine specific gravity was <1.03 (32) and a flexible, sterile, single-use rectal thermistor (Henleys Medical Supplies Ltd., Herts, UK) was self-inserted 10 cm beyond the anal sphincter to measure *T*_re_. A pre-exercise nude body mass was recorded using a digital platform scale (model 703; Seca, Hamburg, Germany) and skin thermistors were attached on the right side of the body for the determination of mean skin temperature (*T*_sk_), as previously described (33). Mean body temperature (*T*_b_) was estimated using the following formula (34):

$$T_{\text{b}}=0.64\times T_{\text{re}}+0.36\times T_{\text{sk}}\text{.}$$

After instrumentation, participants rested for a further 30 min in temperate conditions (19°C, 45% relative humidity) to establish baseline measures. Body surface area (*A*_D_) by the Du Bois equation (35), and V̇o_2_ and respiratory exchange ratio (RER) from a 60-s expired gas collection by Douglas bag method between 29 and 30 min of seated rest were used to estimate resting metabolic heat production (*H*) as follows (36):

$$H\left(\text{W}\cdot {\text{m}}^{-\text{2}}\right)=\left[0.\text{23}\left(\text{RER}\right)+0.\text{77}\right]\times \left[\text{5}.\text{873}\left({\dot{\text{V}}\text{o}}_{\text{2}}\right)\right]\times \left(\text{6}0/A_{\text{D}}\right)\text{.}$$

At 0945, dressed in shorts, socks, and trainers, participants entered the environmental chamber (33°C, 40% relative humidity, 0.2 m·s^−1^ wind velocity) to complete a 40-min treadmill run at 65% V̇o_2peak_. *T*_re_, skin temperatures, and heart rate were monitored continuously. Local forearm sweat rate was measured by dew point hygrometry (DS2000; Alpha Moisture Systems, UK). Anhydrous compressed nitrogen at a flow rate of 1 L·min^−1^ was passed through a 5-cm^2^ capsule, affixed to the ventral surface of the lower arm (halfway between the antecubital fossa and carpus). Local forearm sweat rate was calculated as the difference in water content between effluent and influent air, divided by the skin surface area under the capsule (expressed in milligrams per square centimeter per minute). *T*_re_ at sweating onset was determined by plotting the relationship between local forearm sweat rate and *T*_re_ (recorded at 20-s intervals) before using segmented linear regression to identify the breakpoint in the two line segments (37). Rating of perceived exertion (RPE) (38), thermal sensation (TS) (39), V̇o_2_, and RER (40) were recorded every 10 min. On completion of the exercise, participants rested for 20 min in temperate conditions (19°C, 45% relative humidity), during which they completed a modified Stroop test (41) to assess cognitive function as a marker of overreaching (30), and provided a nude body mass to estimate whole-body sweat rate.

Participants then re-entered the environmental chamber and completed a time to exhaustion (TTE) on a motorized treadmill at 65% V̇o_2peak_. Participants were instructed to “run for as long as possible.” TTE was terminated when participants stopped running owing to volitional exhaustion, thermal discomfort, or when *T*_re_ exceeded 39.5°C. No fluids were consumed, no feedback was provided, and *T*_re_ and heart rate were monitored continuously. After the cessation of exercise, capillary blood lactate concentration was assessed (Lactate Pro 2, Arkray, Australia) as a marker of overreaching (42, 43). Participants were provided with a bolus of water and were free to leave the laboratory when *T*_re_ ≤ 38.5°C.

### Daily Intervention

All participants in *part 1* and *part 2* completed 12 and 6 days of their assigned intervention, respectively. During the intervention, participants were instructed to consume their normal diet and fluid intake, including caffeine and alcohol (≤3 units/day). Participants arrived at the laboratory each day between 0600 and 1300. Before exercise, a nude body mass was taken, and a rectal thermistor and heart rate monitor were fitted. After instrumentation, participants completed a 15-min seated rest in temperate conditions (19°C, 45% relative humidity) to establish baseline measures, before commencing their assigned intervention protocol. A bolus of water (5 mL·kg^−1^ of nude body mass) was consumed during the first 20 min of exercise.

Participants assigned to HWI completed a 40-min treadmill run dressed in shorts, socks, and trainers at a speed equivalent to their 65% V̇o_2peak_ (9.1 ± 1.6 km·h^−1^) in temperate conditions (19°C, 45% relative humidity, 0.2 m·s^−1^ wind velocity). After exercise (2–3 min transition), dressed in shorts, participants began a semirecumbent ≤40-min HWI (40°C) to the neck, as previously described (6). Participants assigned to EHA completed a ≤60-min treadmill run at a speed equivalent to their 65% V̇o_2peak_ (9.1 ± 1.1 km·h^−1^) in an environmental chamber (33°C, 40% relative humidity, 0.2 m·s^−1^ wind velocity). Participants assigned to CON completed a daily submaximal treadmill run equivalent to 65% V̇o_2peak_ and work matched to EHA (8.8 ± 0.9 km·h^−1^) in temperate conditions (19°C, 45% relative humidity, 0.2 m·s^−1^ wind velocity). Owing to the nature of these interventions, it was not possible to blind the participants. In *part 1*, to maintain the endogenous thermal stimulus for adaptation after the first six intervention sessions (*days 1–3* and *days 6–8*, Fig. 1), maximum immersion (HWI) and exercise duration (EHA and CON) increased by 25%, as of the seventh intervention session (*days 11–16*, intervention sessions 7–12), to ≤50 min and ≤75 min, respectively. All intervention sessions were terminated if the maximal immersion/exercise duration was reached, at the participant’s volition, or if *T*_re_ exceeded 39.5°C. Upon removal from the hot water/environmental chamber, participants rested in a seated position for 5 min in a temperate laboratory, were provided with a bolus of water, and were free to leave the laboratory when *T*_re_ ≤ 38.5°C.

### Blood Sample Collection and Analysis

Venous blood samples were collected from an antecubital vein without stasis into two 6-mL EDTA vacutainers (BD, Oxford, UK). Aliquots of whole blood were used for the immediate determination of hemoglobin in duplicate (Hemocue, Sheffield, UK) and hematocrit in triplicate using a microcentrifuge and microhematocrit reader (Hawksley & Sons Limited, Lancing, UK). The change in plasma volume was estimated by correcting the initial plasma volume at PRE for the percentage change in plasma volume (%ΔPV) at POST3, POST6, and POST12, as previously described (44). The remaining whole blood was then centrifuged, and the plasma frozen at −80°C for later analysis.

Plasma concentrations of free and total triiodothyronine (T3) and thyroxine (T4) were measured in duplicate by ELISA (free T3: Cat. No. RE55231, detection limit: 0.1 pmol·L^−1^; free T4: Cat. No. RE55241, detection limit: 0.6 pmol·L^−1^; total T3: Cat. No. RE55251, detection limit: 0.2 nmol·L^−1^; total T4: Cat. No. RE55261, detection limit: 0.1 nmol·L^−1^; IBL International, Hamburg, Germany). The intra-assay coefficient of variation for duplicates were free T3, 5.1%; free T4, 2.6%; total T3, 5.6%; total T4, 5.9%. Thyroid hormone concentrations were adjusted for plasma volume changes using the following formula (45):

Corrected value=Uncorrected value×[(100+%ΔPV)/100]

### Statistical Analysis

Data were analyzed using SPSS version 27 (IBM Corporation) or GraphPad Prism Version 9.1 (GraphPad Software, Inc. La Jolla, CA). All data were checked for normality and sphericity; plasma free T4 data were reciprocal transformed to address statistical assumptions of sphericity. Data are presented as untransformed mean and SD unless otherwise stated, and statistical significance was accepted at *P* < 0.05. In *part 1*, the mean daily endogenous thermal stimulus and external work during HWI, EHA, and CON were compared using a two-way mixed model ANOVA. A two-way mixed-model ANCOVA, with baseline (PRE) as the covariate, was used to detect differences in heat acclimation adaptations, endurance capacity, overreaching markers, and plasma thyroid hormone concentrations after 3, 6, and 12 days of HWI, EHA, or CON. Bonferroni-adjusted pairwise comparisons were used where appropriate to determine where differences occurred. The size of the between-intervention differences was calculated using Cohen’s *d* effect size with values greater than 0.2, 0.5, and 0.8 representing small, medium, and large effects, respectively (46). In *part 2*, the mean daily endogenous thermal stimulus and external work during HWI and CON were compared using *t* tests, and a one-way ANCOVA was used to detect differences in heat acclimation adaptations and plasma thyroid hormone concentrations after 6 days of HWI or CON. Bonferroni-adjusted pairwise comparisons were used where appropriate to determine where differences occurred. Pearson’s correlations determined the strength of the relationship between the endogenous thermal stimulus, changes in resting *T*_re_ and plasma thyroid hormone concentrations after 12 days of heat acclimation by HWI and EHA. Pearson correlation coefficients of 0.00–0.19 were regarded as very weak, 0.20–0.39 as weak, 0.40–0.59 as moderate, and 0.60–0.79 as strong relationships (47).

## RESULTS

### Part 1 Daily Intervention Thermal Stimulus and External Work

Throughout the 12-day intervention, the daily endogenous thermal stimulus for adaptation was similar between HWI and EHA (Table 2; all *P* ≥ 0.407), but lower in CON (*P* < 0.001); there were no main effects of time or interaction effects (*P* ≥ 0.252). The daily endogenous thermal stimulus was maintained throughout the 12 days by an increase in mean daily immersion on HWI (*days 1*–*3*, 33 ± 4 min; *days 6*–*8*, 35 ± 5 min; *days 11*–*16*, 39 ± 5 min, *P* < 0.001) and an increase in exercise duration on EHA (*days 1*–*3*, 51 ± 9 min; *days 6*–*8*, 55 ± 8 min; *days 11*–*16*, 61 ± 11 min, *P* < 0.001). The similar daily thermal stimulus during HWI and EHA was achieved with a lower mean daily external work in HWI than EHA (Table 2; *P* = 0.006), and mean daily external work also tended to be lower in HWI than CON (*P* = 0.053).

**Table 2.** Daily endogenous thermal stimulus and external work during temperate exercise control, exercise heat acclimation, and post-exercise hot water immersion interventions

	*Days 1–3*			*Days 6–8*			*Days 11–16*		
	CON	EHA	HWI	CON	EHA	HWI	CON	EHA	HWI
Duration *T*_re_ ≥ 38.5°C, min	7 ± 12	35 ± 14††	36 ± 5††	8 ± 12	38 ± 11††	38 ± 6††	7 ± 10	38 ± 13††	39 ± 8††
AUC, °C·min^−1^	1 ± 3	17 ± 10††	17 ± 5††	2 ± 4	16 ± 8††	18 ± 4††	1 ± 1	12 ± 6††	20 ± 6††
End intervention *T*_re_, °C	38.24 ± 0.34	39.17 ± 0.28††	39.24 ± 0.16††	38.22 ± 0.46	39.11 ± 0.22††	39.27 ± 0.14††	38.23 ± 0.22	38.99 ± 0.25††	39.31 ± 0.18††
External work, km	7.4 ± 1.1	7.7 ± 1.6	6.1 ± 1.1‡	7.6 ± 1.7	8.1 ± 1.7	6.1 ± 1.1‡	8.7 ± 1.7	9.0 ± 1.5	6.1 ± 1.1‡

Data are represented as means ± SD of *days 1–3*, *days 6–8*, and *days 11–16*. *n* = 7 participants in each group (*part 1*). Analyzed by two-way mixed model ANOVA;

††group difference to CON, *P* < 0.01; ‡group difference to EHA, *P* < 0.05. AUC, area under the curve for *T*_re_ >38.5°C; CON, temperate exercise control; EHA, exercise heat acclimation; HWI, postexercise hot water immersion, *T*_re_, rectal core temperature.

### Part 1 Experimental Trials

Before the experimental trial, standardization ensured sleep duration (6 ± 1 h, *P* ≥ 0.184) and hydration status, as assessed by urine specific gravity (1.020 ± 0.007, *P* ≥ 0.268), were similar between the interventions, as evidenced by no main effects of group or time, and no interaction effects.

#### Thermal responses at rest in temperate conditions.

**Figure 3.**
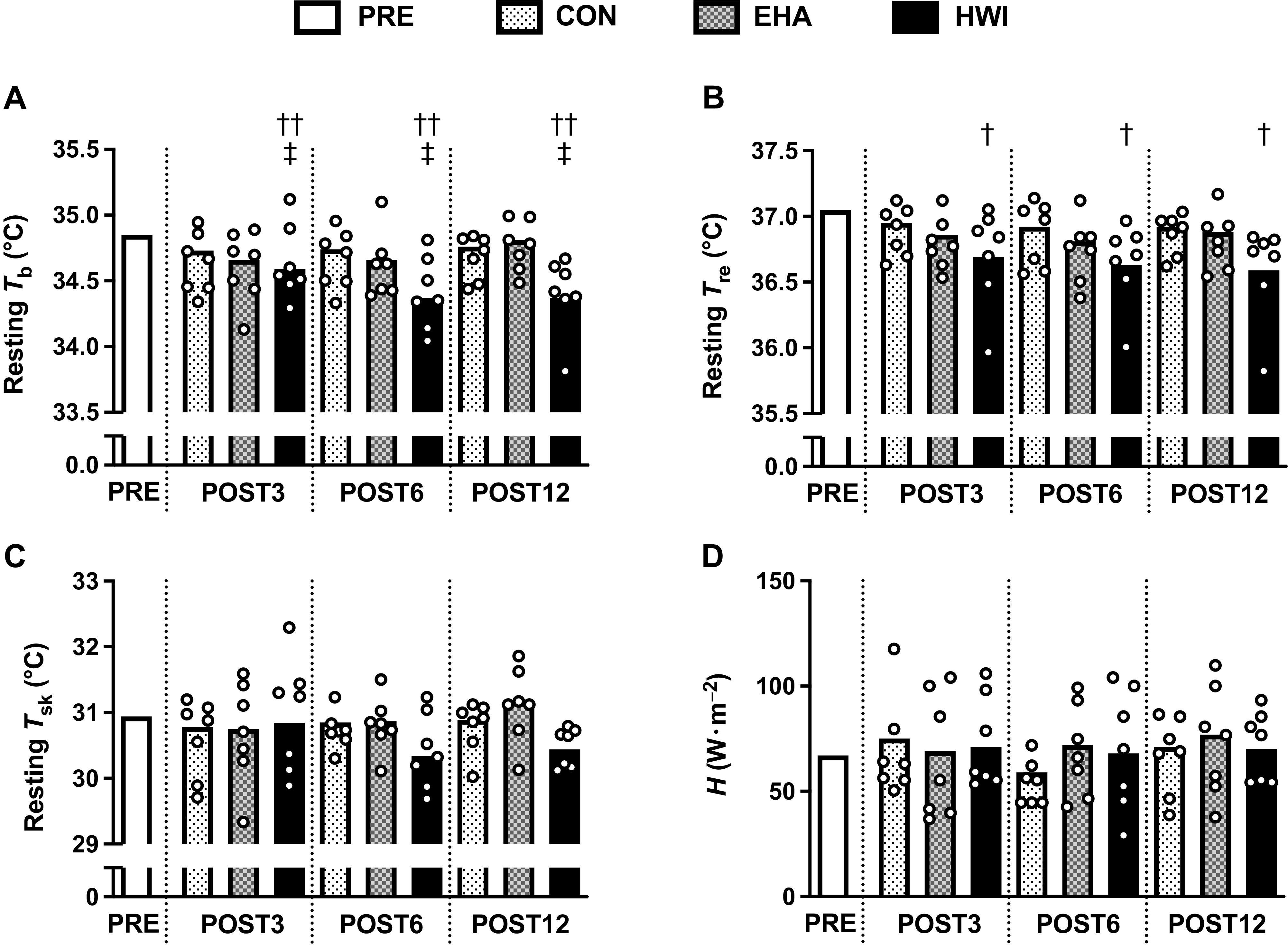
Influence of 3 (POST3), 6 (POST6), and 12 days (POST12) of a temperate exercise control (CON, *n* = 7 participants), exercise heat acclimation (EHA, *n* = 7 participants), or post-exercise hot water immersion (HWI, *n* = 7 participants) on resting mean body temperature (*T*_b_, *A*), rectal core temperature (*T*_re_, *B*), mean skin temperature (*T*_sk_, *C*), and metabolic heat production (*H*, *D*) in temperate conditions (19°C, 45% relative humidity). Bars represent baseline-adjusted means; circles represent individual participant responses. Analyzed by two-way mixed model ANCOVA, with baseline (PRE) as the covariate and Bonferroni-adjusted pairwise comparisons; †HWI lower than CON, *P* < 0.05; ††HWI lower than CON, *P* < 0.01; ‡HWI lower than EHA, *P* < 0.05.

Thermal responses at rest in temperate conditions were different between interventions after 12 days. Resting *T*_b_ was lower after HWI than EHA (Fig. 3*A*, *P* = 0.009, *d* = 1.86) and CON (*P* = 0.005, *d* = 2.04). Resting *T*_b_ was not different between EHA and CON over the 12 days (*P* = 1.000, *d* = 0.20). The average reduction in resting *T*_b_ over the 12 days was −0.41 ± 0.15°C for HWI, −0.14 ± 0.14°C for EHA, and −0.11 ± 0.15°C for CON. Resting *T*_re_ was lower after HWI (Fig. 3*B*, −0.41 ± 0.15°C) than CON (−0.12 ± 0.15°C, *P* = 0.007, *d* = 1.93) but not EHA (−0.20 ± 0.15°C, *P* = 0.061, *d* = 1.37). Resting *T*_re_ was not different between EHA and CON over the 12 days (*P* = 0.936, *d* = 0.56). Conversely, there were no differences between interventions for resting *T*_sk_ (Fig. 3*C*), resting *T*_re_ − *T*_sk_ gradient, resting *H* (Fig. 3*D*), or plasma volume (all *P* ≥ 0.096; Table 3).

**Table 3.** Change from baseline in heat acclimation adaptations at rest (19 °C, 45% RH) and during 40-min submaximal exercise in the heat (33 °C, 40% relative humidity) after 3, 6 and 12 days of temperate exercise control, exercise heat acclimation, and post-exercise hot water immersion interventions

	CON			EHA			HWI		
	POST3	POST6	POST12	POST3	POST6	POST12	POST3	POST6	POST12
*Rest*									
Resting *T*_b_, °C	−0.12 ± 0.20	−0.11 ± 0.22	−0.09 ± 0.20	−0.19 ± 0.20	−0.18 ± 0.21	−0.03 ± 0.20	−0.26 ± 0.20††,‡‡	−0.48 ± 0.22††,‡‡	−0.48 ± 0.20††,‡‡
Resting *T*_re_, °C	−0.10 ± 0.19	−0.13 ± 0.18	−0.13 ± 0.19	−0.19 ± 0.19	−0.24 ± 0.18	−0.17 ± 0.19	−0.35 ± 0.19††	−0.41 ± 0.18††	−0.46 ± 0.19††
Resting *T*_sk_, °C	−0.17 ± 0.63	−0.09 ± 0.36	−0.05 ± 0.42	−0.19 ± 0.62	−0.07 ± 0.36	−0.23 ± 0.41	−0.10 ± 0.63	−0.60 ± 0.36	−0.50 ± 0.42
Resting *H*, W·m^−2^	7 ± 20	−9 ± 15	4 ± 17	2 ± 20	4 ± 15	9 ± 17	3 ± 21	0 ± 15	3 ± 17
Plasma volume, %	3 ± 7	3 ± 5	2 ± 7	3 ± 7	6 ± 5	5 ± 7	1 ± 7	4 ± 5	3 ± 7
*Submaximal exercise*									
End-exercise *T*_b_, °C	−0.27 ± 0.24	−0.36 ± 0.24	−0.52 ± 0.25	−0.33 ± 0.25	−0.44 ± 0.25	−0.62 ± 0.26	−0.39 ± 0.25	−0.58 ± 0.25	−0.83 ± 0.26
End-exercise *T*_re_, °C	−0.21 ± 0.23	−0.36 ± 0.21	−0.41 ± 0.20	−0.32 ± 0.24	−0.33 ± 0.21	−0.44 ± 0.21	−0.32 ± 0.23†	−0.56 ± 0.21†	−0.64 ± 0.20†
Δ*T*_re_ during exercise, °C	−0.10 ± 0.26	−0.22 ± 0.29	−0.28 ± 0.29	−0.16 ± 0.28	−0.09 ± 0.30	−0.29 ± 0.30	−0.06 ± 0.27	−0.15 ± 0.30	−0.18 ± 0.30
*T*_re_ at sweating onset, °C	−0.15 ± 0.16	−0.15 ± 0.19	−0.18 ± 0.15	−0.19 ± 0.17	−0.29 ± 0.19	−0.19 ± 0.15	−0.30 ± 0.17††,‡	−0.47 ± 0.19††,‡	−0.50 ± 0.15††,‡
Whole-body sweat rate, L·h^−1^	−0.05 ± 0.09	−0.05 ± 0.06	0.07 ± 0.11	0.06 ± 0.09†	0.04 ± 0.06†	0.09 ± 0.10†	0.08 ± 0.09††	0.08 ± 0.06††	0.10 ± 0.10††
End-exercise *T*_sk_, °C	−0.38 ± 0.49	−0.38 ± 0.46	−0.73 ± 0.54	−0.39 ± 0.50	−0.66 ± 0.47	−0.95 ± 0.55	−0.50 ± 0.52	−0.60 ± 0.48	−1.15 ± 0.57
End-exercise HR, beats·min^−1^	−8 ± 5	−12 ± 7	−14 ± 8	−12 ± 5	−15 ± 7	−20 ± 8	−11 ± 5	−17 ± 7	−20 ± 8
Mean V̇o_2_, L·min^−1^	−0.10 ± 0.13	−0.10 ± 0.15	−0.16 ± 0.13	−0.01 ± 0.13	0.00 ± 0.15	−0.06 ± 0.13	−0.04 ± 0.13	−0.04 ± 0.14	−0.06 ± 0.12
Mean RER	−0.01 ± 0.04	−0.02 ± 0.03	−0.03 ± 0.04	−0.02 ± 0.04	−0.02 ± 0.03	−0.01 ± 0.04	−0.02 ± 0.04	−0.02 ± 0.03	−0.02 ± 0.04
End-exercise RPE, 6–20 scale	0 ± 2	0 ± 1	0 ± 2	−1 ± 2	−1 ± 1	−1 ± 2	−2 ± 2†	−2 ± 1†	−2 ± 2†
End-exercise TS, 1–13 scale	0 ± 1	0 ± 1	0 ± 1	0 ± 1	−1 ± 1	−1 ± 1	−1 ± 1†	−1 ± 1†	−1 ± 1†

Data are baseline-adjusted mean change ± SD after 3 (POST3), 6 (POST6), and 12 days (POST12). *n* = 7 participants in each group (*part 1*). CON, temperate exercise control; EHA, exercise heat acclimation; HWI, post-exercise hot water immersion; *H*, metabolic heat production; HR, heart rate; RER, respiratory exchange ratio; RPE, rating of perceived exertion; TS, thermal sensation; *T*_b_, mean body temperature; *T*_re_, rectal core temperature; *T*_sk_, mean skin temperature. Analyzed by two-way mixed model ANCOVA, with baseline (PRE) as the covariate and Bonferroni-adjusted pairwise comparisons;

†group difference to CON, *P* < 0.05; ††group difference to CON, *P* < 0.01; ‡group difference to EHA, *P* < 0.05.

#### Thermal and perceptual responses to exercise in the heat.

**Figure 4.**
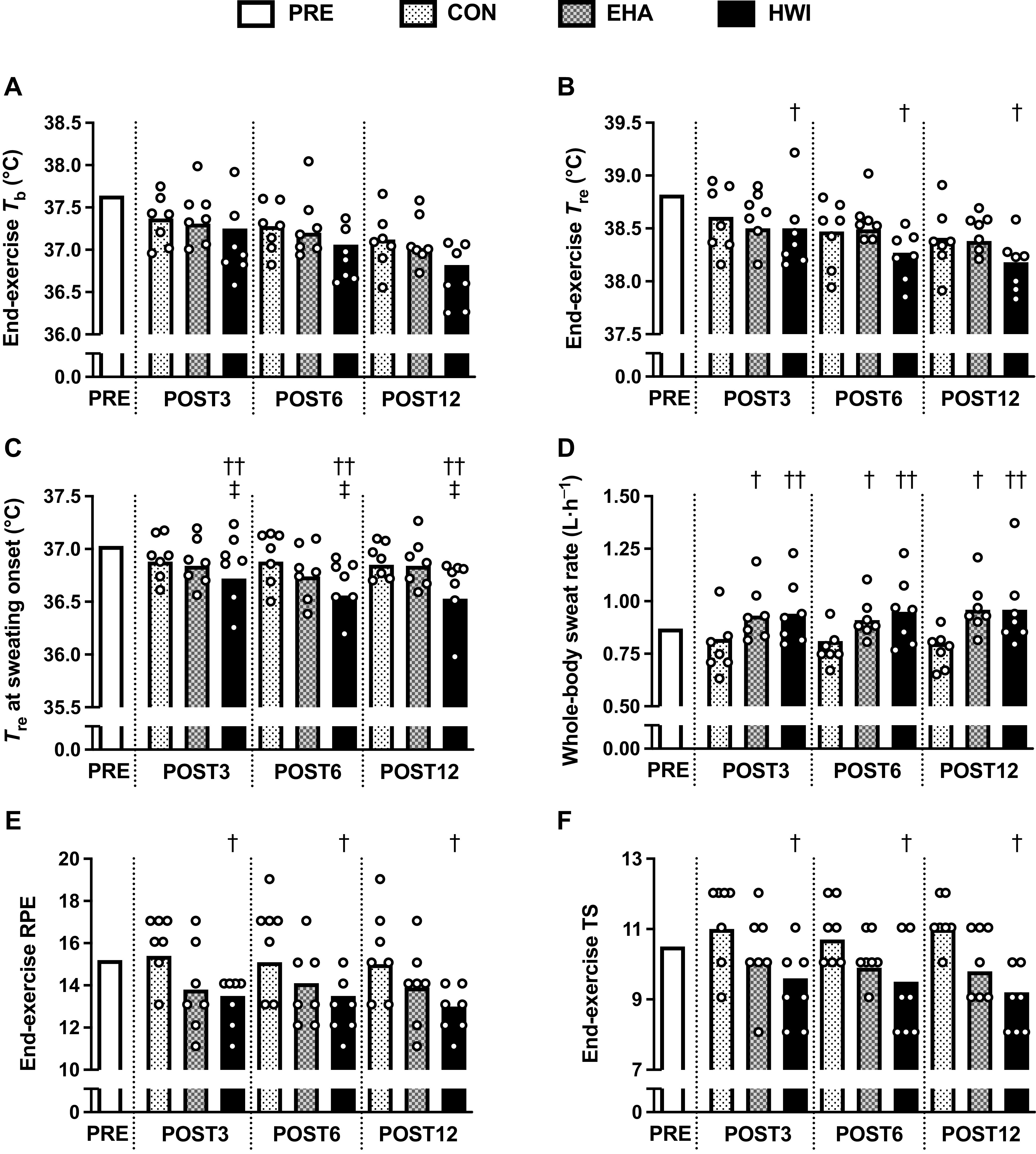
Influence of 3 (POST3), 6 (POST6), and 12 days (POST12) of a temperate exercise control (CON, *n* = 7 participants), exercise heat acclimation (EHA, *n* = 7 participants), or post-exercise hot water immersion (HWI, *n* = 7 participants) on end-exercise mean body temperature (*T*_b_, *A*), end-exercise rectal core temperature (*T*_re_, *B*), *T*_re_ at sweating onset (*C*), whole-body sweat rate (*D*), end-exercise rating of perceived exertion (RPE, *E*), and end-exercise thermal sensation (TS, *F*) in the heat (33°C, 40% relative humidity). Bars represent baseline-adjusted means; circles represent individual participant responses. Analyzed by two-way mixed model ANCOVA, with baseline (PRE) as the covariate and Bonferroni-adjusted pairwise comparisons; †group difference to CON, *P* < 0.05; ††group difference to CON, *P* < 0.01; ‡HWI lower than EHA, *P* < 0.05.

Thermal and perceptual responses to submaximal exercise in the heat were different between the interventions after 12 days. End-exercise *T*_re_ after exercise-heat-stress was lower after HWI (Fig. 4*B*, −0.50 ± 0.19°C) than CON (−0.33 ± 0.13°C; *P* = 0.049, *d* = 1.13) but not EHA (−0.37 ± 0.13°C; *P* = 0.196, *d* = 0.88); no difference was observed between EHA and CON (*P* = 1.000, *d* = 0.30). *T*_re_ at sweating onset was lower after HWI (Fig. 4*C*, −0.43 ± 0.12°C) than EHA (−0.22 ± 0.12°C; *P* = 0.015, *d* = 1.75) and CON (−0.16 ± 0.12°C; *P* = 0.002, *d* = 2.27). Conversely, EHA did not reduce *T*_re_ at sweating onset compared with CON (*P* = 1.000, *d* = 0.52). Whole-body sweat rate was greater after HWI (Fig. 4*D*, +0.08 L·h^−1^; *P* = 0.003, *d* = 2.13) and EHA (+0.06 L·h^−1^; *P* = 0.013, *d* = 1.78) than CON (−0.06 L·h^−1^), but no difference was detected between HWI and EHA (*P* = 1.000, *d* = 0.35). In accordance with thermal adaptations, perceptual responses to exercise-heat-stress were lower after HWI (RPE Fig. 4*E*, −2 ± 1; TS Fig. 4*F*, −1 ± 1) than CON (RPE, 0 ± 1, *P* = 0.036, *d* = 1.57; TS, 0 ± 1, *P* = 0.047, *d* = 1.55) but not EHA (RPE, −1 ± 1, *P* = 0.951, *d* = 0.54; TS, −1 ± 1, *P* = 1.000, *d* = 0.55); no differences were observed between EHA and CON (*P* ≥ 0.157, *d* = 1.07). There were no differences between interventions for the change in *T*_re_ during the 40-min treadmill run in the heat, end-exercise *T*_b_ (Fig. 4*A*), end-exercise *T*_sk_, end-exercise *T*_re_ − *T*_sk_ gradient, end-exercise heart rate, exercising V̇o_2_, or exercising RER (Table 3; all *P* ≥ 0.059). The rate of thermal and perceptual adaptations was not different among HWI, EHA, or CON from POST3 to POST12, as indicated by no interaction effects (all *P* ≥ 0.087). There were also no main effects of time (all *P* ≥ 0.148).

#### Overreaching markers and endurance capacity.

There was no evidence to suggest that 12 days of HWI or EHA induced overreaching to a greater extent than CON, with no interaction effects, main effects of group or time detected for total mood disturbance, energy index, Stroop reaction time, Stroop accuracy, sleep duration, or sleep efficiency (Table 4; all *P* ≥ 0.190). Five participants were removed from the TTE endurance capacity test analysis owing to reaching the *T*_re_ ethical cut-off (HWI, *n* = 2); going to the toilet (EHA, *n* = 1); exercise-induced bronchoconstriction (CON, *n* = 1); and an obvious lack of effort without markers of overreaching at rest (CON, *n* = 1). Analysis of the remaining 16 participants (HWI, *n* = 5; EHA, *n* = 6; CON, *n* = 5) who completed the TTE revealed no statistical differences between interventions or across time (Table 4; *P* ≥ 0.219). In addition, no differences were detected between interventions for end-TTE *T*_re_, end-TTE heart rate, or end-TTE blood lactate concentration (Table 4; all *P* ≥ 0.198).

**Table 4.** Change from baseline in markers of overreaching and endurance capacity in the heat (33°C, 40% relative humidity) after 3, 6 and 12 days of temperate exercise control, exercise heat acclimation, and post-exercise hot water immersion interventions

	CON			EHA			HWI		
	POST3	POST6	POST12	POST3	POST6	POST12	POST3	POST6	POST12
*Markers of overreaching*									
Total mood disturbance	5 ± 10	2 ± 12	2 ± 10	5 ± 10	7 ± 12	2 ± 10	4 ± 10	4 ± 12	2 ± 10
Energy index	−3 ± 4	−2 ± 6	−3 ± 5	−3 ± 4	−5 ± 6	−3 ± 5	−2 ± 4	−4 ± 6	−3 ± 5
Stroop reaction time, ms	−29 ± 58	−25 ± 40	−11 ± 64	−13 ± 58	−32 ± 40	−15 ± 63	−16 ± 62	−18 ± 43	−28 ± 68
Stroop accuracy, %	0 ± 2	−1 ± 3	1 ± 4	−1 ± 3	−1 ± 3	−2 ± 4	2 ± 3	1 ± 3	0 ± 4
Sleep duration, h	6 ± 1	6 ± 1	6 ± 1	6 ± 1	6 ± 1	6 ± 1	6 ± 1	6 ± 1	6 ± 1
Sleep efficiency, %	0 ± 9	−2 ± 7	−1 ± 8	−6 ± 9	−5 ± 7	1 ± 8	−2 ± 9	2 ± 7	−2 ± 8
*Endurance capacity*									
TTE, s	−27 ± 676	75 ± 808	212 ± 991	101 ± 627	539 ± 749	323 ± 919	321 ± 743	686 ± 888	1,030 ± 1,089
End-TTE *T*_re_, °C	−0.14 ± 0.30	−0.24 ± 0.34	−0.32 ± 0.47	−0.20 ± 0.31	−0.06 ± 0.34	−0.29 ± 0.47	−0.20 ± 0.31	−0.03 ± 0.34	−0.25 ± 0.47
End-TTE HR beats·min^−1^	−8 ± 8	−10 ± 7	−16 ± 10	−10 ± 8	−12 ± 7	−20 ± 10	−8 ± 8	−10 ± 7	−14 ± 10
End-TTE blood lactate, mmol·L^−1^	0.2 ± 1.4	−0.1 ± 0.7	0.2 ± 0.6	0.5 ± 1.3	−0.2 ± 0.7	−0.9 ± 0.6	−0.2 ± 1.3	−0.1 ± 0.7	0.2 ± 0.6

Data are baseline-adjusted mean change ± SD after 3 (POST3), 6 (POST6), and 12 days (POST12). *n* = 21, except *n* = 16 participants for TTE (*part 1*). CON, temperate exercise control; EHA, exercise heat acclimation; HR, heart rate; HWI, post-exercise hot water immersion; *T*_re_, rectal core temperature; TTE, time to exhaustion. Analyzed by two-way mixed model ANCOVA, with baseline (PRE) as the covariate.

#### Thyroid hormones.

**Figure 5.**
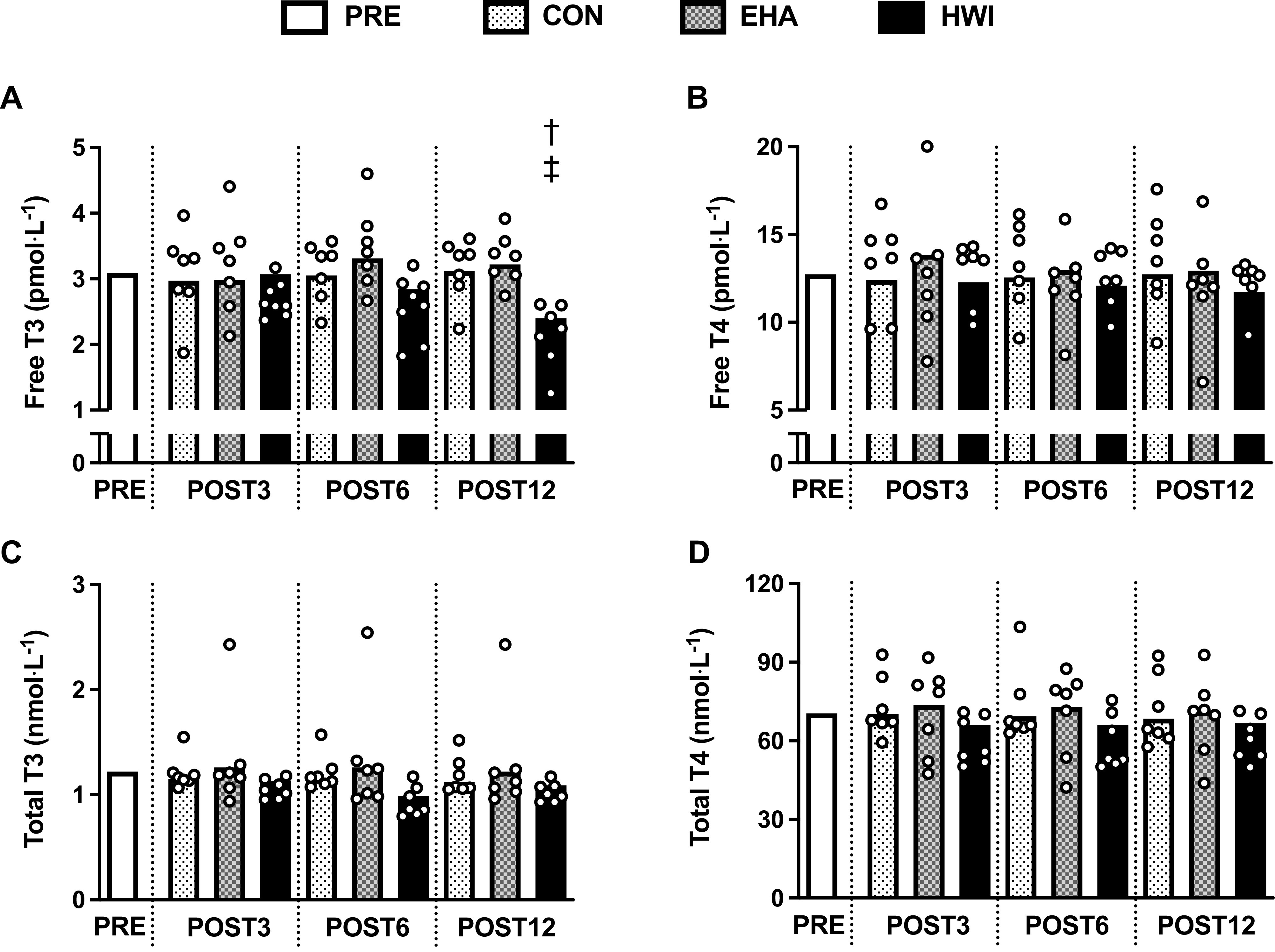
Influence of 3 (POST3), 6 (POST6), and 12 days (POST12) of a temperate exercise control (CON, *n* = 7 participants), exercise heat acclimation (EHA, *n* = 7 participants), or post-exercise hot water immersion (HWI, *n* = 7 participants) on plasma concentrations of free triiodothyronine (T3, *A*), free thyroxine (T4, *B*), total T3 (*C*), and total T4 (*D*). Bars represent baseline-adjusted means; circles represent individual participant responses. Analyzed by two-way mixed model ANCOVA, with baseline (PRE) as the covariate and Bonferroni-adjusted pairwise comparisons; †HWI lower than CON, *P* < 0.05; ‡HWI lower than EHA, *P* < 0.05.

Twelve days of HWI elicited a reduction in thyroid hormones, evidenced by an interaction effect for plasma concentrations of free T3 (*P* = 0.006). Follow-up analyses showed that free T3 was lower after 12 days of HWI (−23%) than EHA (+4%, *P* = 0.008) and CON (+1%, *P* = 0.015; Fig. 5*A*). No differences were detected for free T3 between EHA and CON (*P* = 1.000). Conversely, there were no interaction effects or main effects of group or time detected for plasma concentrations of free T4 (*P* ≥ 0.148, Fig. 5*B*), total T3 (*P* ≥ 0.057, Fig. 5*C*), or total T4 (*P* ≥ 0.156, Fig. 5*D*).

### Part 2 Daily Intervention Thermal Stimulus and External Work

All 48 participants completed 6 days of their assigned intervention. The HWI intervention caused a greater daily endogenous thermal stimulus than CON as indicated by greater daily duration *T*_re_ > 38.5°C (HWI, 41 ± 13 min; CON, 7 ± 8; *P* < 0.001), area under the curve (AUC) for *T*_re_ > 38.5°C (HWI, 23 ± 10 °C·min^−1^; CON, 1 ± 2 °C·min^−1^; *P* < 0.001), and end-intervention *T*_re_ (HWI, 39.3 ± 0.2°C; CON, 38.3 ± 0.4°C; *P* < 0.001). Daily external work was similar between HWI and CON (HWI, 7.0 ± 1.1 km; CON 7.3 ± 1.3 km; *P* = 0.065).

### Part 2 Experimental Trials

#### Thermal responses at rest in temperate conditions.

**Figure 6.**
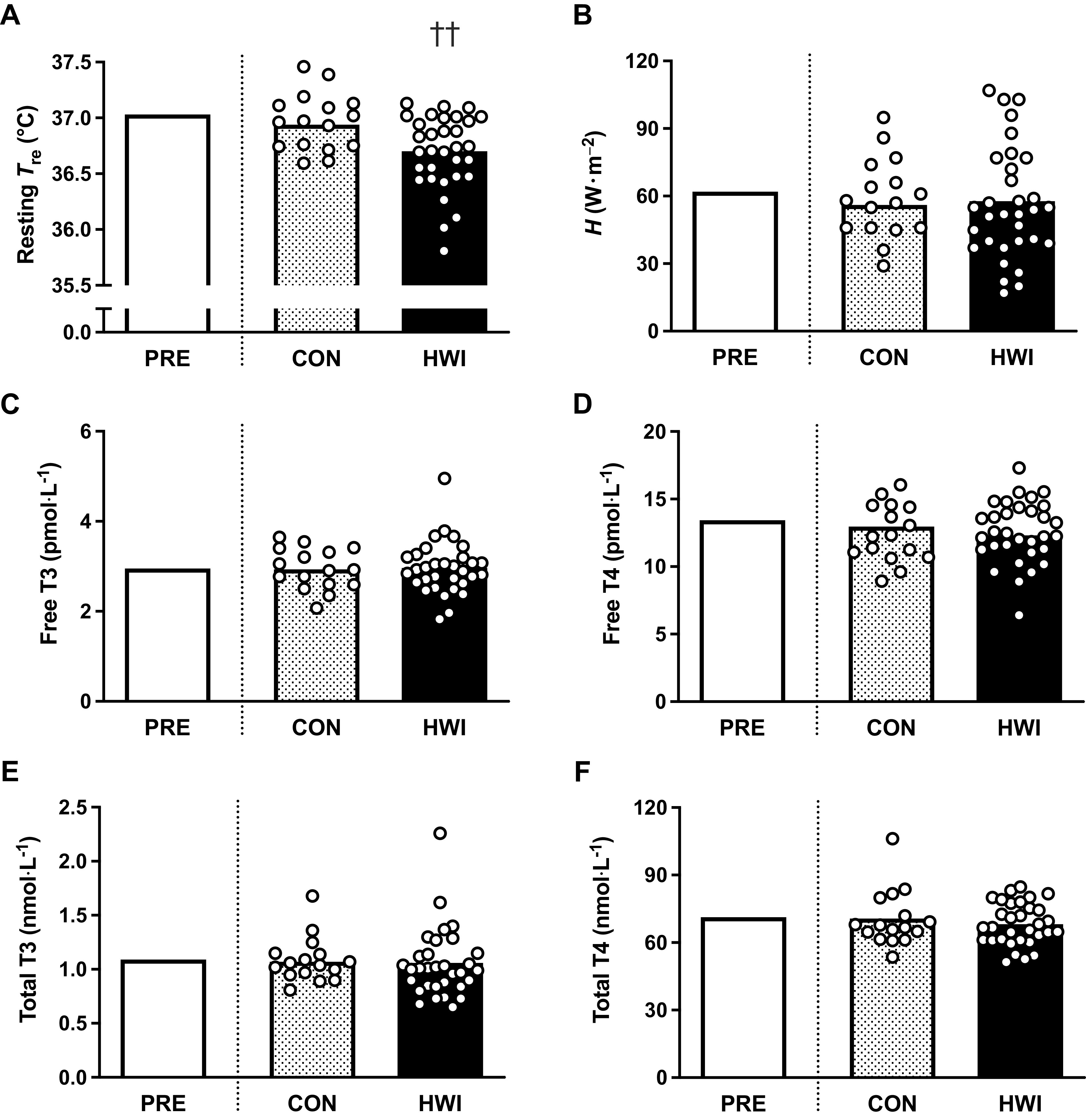
Influence of 6 days of a temperate exercise control (CON, *n* = 16 participants) or post-exercise hot water immersion (HWI, *n* = 32 participants) on resting rectal core temperature (*T*_re_, *A*), resting metabolic heat production (*H*, *B*), and resting plasma concentrations of free triiodothyronine (T3, *C*), free thyroxine (T4, *D*), total T3 (*E*), and total T4 (*F*). Bars represent baseline-adjusted means; circles represent individual participant responses. Analyzed by one-way ANCOVA, with baseline (PRE) as the covariate; ††HWI lower than CON, *P* < 0.01.

Resting *T*_b_ was lower after 6 days of HWI than CON (HWI, −0.31 ± 0.32°C; CON, −0.04 ± 0.32°C; *P* = 0.009). In accordance with resting *T*_b_, resting *T*_re_ was also lower after 6 days of HWI than CON (HWI, −0.33 ± 0.20°C; CON, −0.09 ± 0.21°C; *P* = 0.001, Fig. 6*A*). Conversely, no differences were detected for resting *T*_sk_ (*P* = 0.083), resting *T*_re_ − *T*_sk_ gradient (*P* = 0.509), resting *H* (*P* = 0.711, Fig. 6*B*), or plasma volume (*P* = 0.387).

#### Thermal and perceptual responses to exercise in the heat.

Compared with CON, 6 days of HWI also resulted in a lower end-exercise *T*_b_ (HWI, −0.54 ± 0.24°C; CON, −0.18 ± 0.24°C; *P* < 0.001), end-exercise *T*_re_ (HWI, −0.42 ± 0.24°C; CON, −0.13 ± 0.24°C; *P* < 0.001), *T*_re_ at sweating onset (HWI, −0.31 ± 0.20°C; CON, −0.08 ± 0.19°C; *P* = 0.01), end-exercise *T*_sk_ (HWI, −0.74 ± 0.54°C; CON, −0.30 ± 0.54°C; *P* < 0.001), end-exercise RPE (HWI, −1 ± 1; CON, 0 ± 1; *P* = 0.010), and end-exercise TS (HWI, −1 ± 1; CON, 0 ± 1; *P* = 0.003). No differences were detected for whole-body sweat rate (*P* = 0.228).

#### Thyroid hormones.

**Figure 7.**
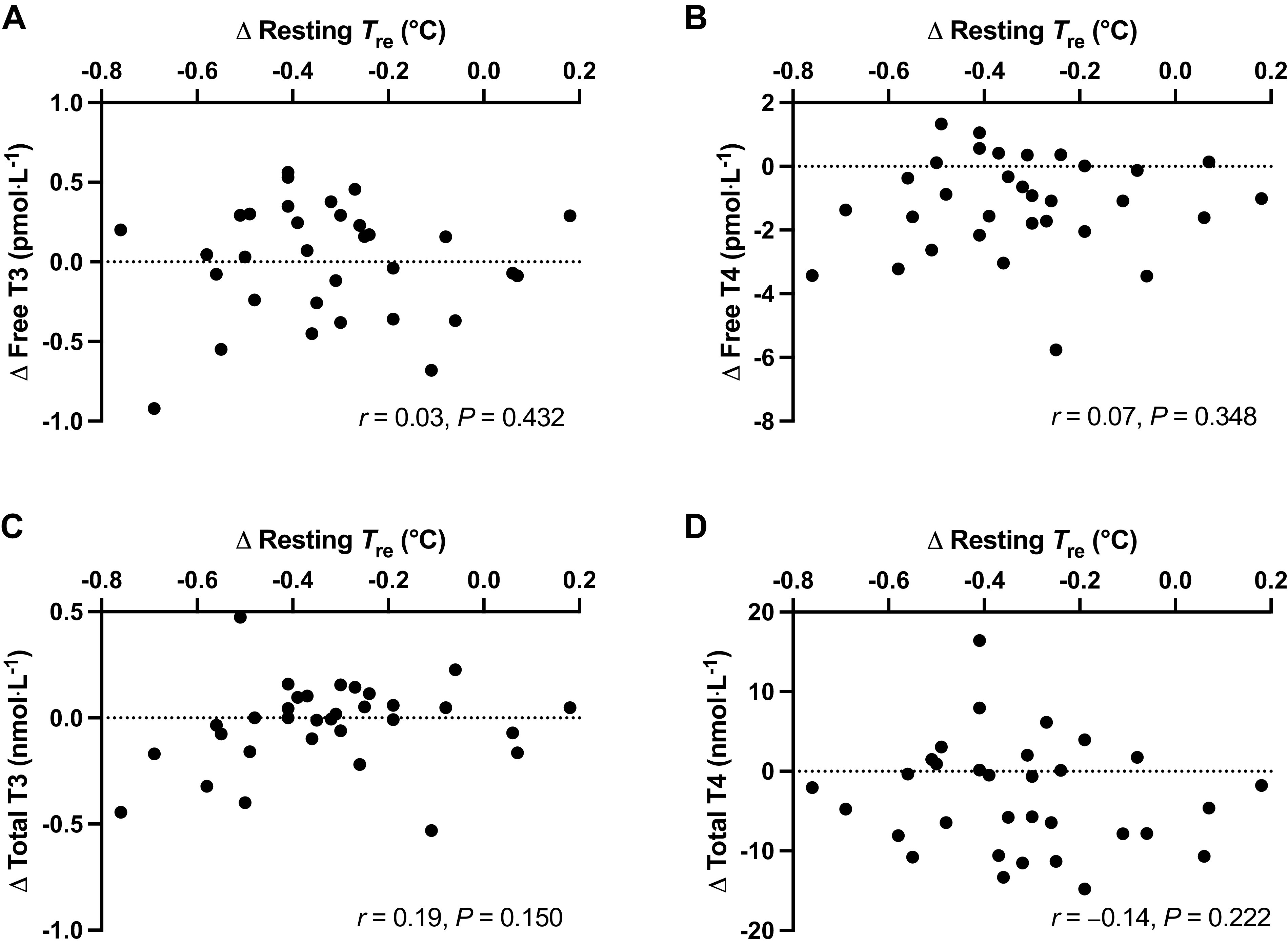
Relationships between the changes in resting core temperature (*T*_re_) and plasma concentrations of thyroid hormones free triiodothyronine (T3, *A*), free thyroxine (T4, *B*), total T3 (*C*), and total T4 (*D*) after 6 days of post-exercise hot water immersion (*n* = 32 participants). Analyzed by Pearson's correlation.

Despite 6 days of HWI causing pronounced heat acclimation adaptations, including reductions in *T*_b_ and *T*_re_ at rest and during exercise in the heat, no differences between HWI and CON were detected in resting plasma thyroid hormone concentrations; free T3 (HWI, 0 ± 12%; CON, −1 ± 12%; *P* = 0.802; Fig. 6*C*), free T4 (HWI, −8 ± 10%; CON, −3 ± 10%; *P* = 0.108; Fig. 6*D*), total T3 (HWI, −3 ± 10%; CON, −2 ± 17%; *P* = 0.873; Fig. 6*E*), or total T4 (HWI, −4 ± 8%; CON, −1 ± 8%; *P* = 0.180; Fig. 6*F*). Moreover, after 6 days of HWI, only weak nonsignificant relationships were observed between the reduction in resting *T*_b_, resting *T*_re_ (Fig. 7), resting *T*_sk_, resting *T*_re_ − *T*_sk_ gradient, end-exercise *T*_b_, end-exercise *T*_re_, *T*_re_ at sweating onset, end-exercise *T*_sk_ or end-exercise TS and changes in free T3 (*r* ≤ 0.21, *P* ≥ 0.269), free T4 (*r* ≤ 0.20, *P* ≥ 0.274), total T3 (*r* ≤ 0.31, *P* ≥ 0.086), and total T4 (*r* ≤ 0.24, *P* ≥ 0.193).

## DISCUSSION

This research is the first to compare hallmark heat acclimation adaptations, endurance capacity, and overreaching markers after 12 days of HWI and EHA with work-matched CON. This study is also the first in humans to examine the potential role of plasma thyroid hormone changes as a mechanism for thermal adaptations after heat acclimation, specifically HWI heat acclimation. The three primary findings of this research conducted in recreationally active men are the following: *1*) In line with our hypothesis, HWI elicited a larger and a greater number of thermal adaptations, and reductions in perceived strain during exercise-heat-stress compared with CON and EHA over the 12-day interventions. Conventional EHA provided only modest further heat acclimation benefits to work-matched CON, *2*) Contrary to our hypothesis, and previous literature examining short-term heat acclimation (12), there was no evidence to suggest that HWI or EHA induced overreaching risk more than with exercise in temperate conditions, and *3*) Also contrary to our hypothesis, changes in plasma thyroid hormone concentrations were not significantly associated with changes in thermal adaptations over the 12 days of HWI, indicating that a reduction in thyroid hormones is unlikely the cause of the pronounced reduction in resting and end-exercise core temperature observed consistently after HWI heat acclimation. Instead, we provide evidence that the reduction in core temperature elicited by postexercise HWI intervention represents the establishment of a new lower thermal balance point (17).

Previous research has demonstrated that short-term (<7 days) HWI provides beneficial heat acclimation adaptations in comparison with CON and conventional EHA in recreationally active males (6, 9, 13, 14). The current study furthers this work by showing that 12 days of HWI heat acclimation led to more pronounced resting and exercising thermal adaptations than EHA and CON (Figs. 3 and 4). Resting *T*_b_ and *T*_re_ at sweating onset were lower over the 12-day HWI intervention than the 12-day EHA intervention. Compared with CON, HWI led to a greater number of thermal adaptations than EHA, i.e., HWI reduced resting *T*_b_, resting *T*_re_, end-exercise *T*_re_, *T*_re_ at sweating onset, end-exercise RPE, end-exercise TS, and increased whole-body sweat rate, whereas EHA increased whole-body sweat rate only. The data also suggests that improvements in endurance capacity in the heat may be more readily observed after HWI than EHA, which has practical implications for applied practitioners and coaches. However, due to dropout, future studies are required to confirm (or reject) this preliminary finding. In combination, these findings indicate that HWI leads to larger and more complete heat acclimation than conventional EHA, even when the endogenous thermal stimulus for adaptation is similar. Heat acclimation adaptations developed throughout the 12 days, with the largest proportion of the adaptations occurring within the first 3 days, for example, ∼58% of the 12-day reduction in end-exercise *T*_re_ was observed on *day 3* (Fig. 4*B*). Nevertheless, we observed no further statistically significant thermal benefits or improvements in endurance capacity by extending the 6-day heat acclimation interventions to 12 days. These findings align with the majority of previous studies that show no further thermal adaptations in males after medium- compared with short-term interventions (10, 26, 48, 49), even when a progressive heat acclimation method was used (27). Far less studied is the influence of additional heat acclimation days on exercise performance. In contrast with our findings, previous research showed additional improvements in exercise performance in the heat when extending exercise heat acclimatization from 6 to 14 days (50). The disparity with our findings may be explained by the small sample size for the TTE outcome in the current study and/or by differences in intervention methods and/or participants’ training status (recreationally active vs. competitive) (51).

The change in *T*_re_ during the 40-min submaximal treadmill run in the heat was similar on all interventions; hence, the lower end-exercise *T*_re_ (i.e., lower thermal strain) after HWI can be attributed to larger reductions in resting *T*_re_ than observed after CON. The induction of large reductions in resting *T*_re_ after HWI is likely due to exposure to a large dual thermal stimulus (i.e., maintained elevation in both core and skin temperature), as it is purported to induce a more complete state of heat acclimation (52). We anticipated the larger thermal adaptations from HWI would be associated with larger reductions in thyroid hormone concentrations in accordance with previous literature, which demonstrate a lower core temperature in hypothyroid compared with control rats (23, 24). However, despite large reductions in resting and end-exercise *T*_re_ after 3, 6, and 12 days of HWI, a concomitant reduction in plasma thyroid hormone concentrations (free T3) was only observed after 12 days (Fig. 5*A*). The temporal disconnect and the absence of significant relationships between changes in thyroid hormones and thermal adaptations indicate that circulating thyroid hormone changes are unlikely the cause of short- and medium-term heat acclimation adaptations. Indeed, the change in free T3 observed after 12 days appears a consequence of HWI heat acclimation. We can further refute the notion that HWI heat acclimation reduces core temperature via alterations in thyroid hormones and metabolism as we did not observe differences between interventions or a reduction in resting *H* after HWI (Fig. 3*D* and Fig. 6*B*). The lower resting core temperature after HWI is also unlikely explained by increased heat loss mechanisms as skin temperature, an index of skin blood flow, was not higher after HWI. In fact, a trend (*P* < 0.1) was observed for lower skin temperature after HWI in both *parts 1* and *2* (Fig. 3*C*). The large reduction in resting core temperature observed after HWI heat acclimation may alternatively be explained by the establishment of a new lower thermal balance point (17). In this study, the new lower thermal balance point is indicated by a lower resting whole body temperature with no change in resting core to skin temperature gradient (Fig. 3*A*).

A combined stimulus of exercise and heat stress is generally considered the “gold standard” method for inducing heat acclimation adaptations (53). As expected, we found that conventional EHA caused thermal adaptations in comparison with baseline (end-exercise: *T*_re_ −0.37 ± 0.13°C, Table 3). However, there is a dearth of medium-term heat acclimation studies with an appropriate control intervention; hence, the true effect of conventional exercise heat acclimation is poorly understood. In the current study, the inclusion of a work-matched temperate exercise intervention allowed the independent effectiveness of the exercise and heat stress stimuli to be determined. We found that, aside from an increase in whole-body sweat rate, which was greater after EHA, no additional heat acclimation adaptations existed between EHA and CON. Our findings align with studies that demonstrate aerobic training in temperate conditions initiates adaptations commonly associated with heat acclimation in recreationally active individuals (54–57). These studies suggest it is principally the endogenous heat production incurred during exercise rather than the external environmental temperature that is important for initiating heat acclimation adaptations. When considered together with these investigations, the benefits of conventional exercise-based heat acclimation beyond work-matched exercise in temperate conditions are modest.

Previous research has shown that intensified training during exercise heat acclimation can trigger markers of overreaching including increased perceived fatigue and decreased performance (12). In contrast, we observed no evidence of overreaching after EHA or HWI; a discrepancy that might be explained by the lower exercise intensity and the inclusion of three rest days in our study compared with previous research. More participants did however withdraw with lower limb discomfort (i.e., knee/ankle pain, etc.) in EHA (25%) and CON (25%) than in HWI (7%; Fig. 2); a finding that might be explained by the ∼35% greater external work during EHA and CON interventions than HWI. This finding provides insight into the practical feasibility of these interventions but is difficult to compare with previous research as heat acclimation studies do not often report participant flow and attrition. Based on our findings, athletes and coaches may be more inclined to choose HWI in the knowledge it carries less injury risk than EHA. Although this is a reasonable hypothesis, future studies with adequate sample sizes are required to specifically evaluate the injury and illness risks of heat acclimation.

Athletes and coaches should consider HWI rather than EHA before traveling to hot climates as it leads to a more complete state of heat acclimation, can be incorporated into the post-exercise washing routine, and eliminates the requirement for an increased training load or access to an environmental chamber. These benefits reduce the disruption to normal training compared with conventional exercise-based strategies, which is especially important during tapering in the lead-up to sporting events. Whereas adverse events after HWI, including syncope, have not been observed by us (6, 7, 13, 14), or reported by others (58, 59), practitioners should follow protocol guidelines carefully. In particular, hot water immersions should be terminated at the participant’s volition or if *T*_re_ exceeds 39.5°C rather than attempting to complete 40 min. In our study, this led to a gradual daily increase in hot water immersion duration up to a maximum of 40 min for the first six (*days 1*–*3*, 33 ± 4 min; *days 6*–*8*, 35 ± 5 min), and then 50 min for the seventh to twelfth immersions (*days 11*–*16*, 39 ± 5 min). The current and previous studies demonstrate the effectiveness of HWI to prepare young, healthy, active males (6, 7, 9, 13, 14) and elderly males and females for heat stress (59). Further research is required to confirm that HWI is effective to cause beneficial thermal, perceptual, and performance adaptations in pediatric, female, and older athletic populations. We hypothesize that HWI will be an effective strategy in these populations as Mee et al. (60) demonstrated that combining both active and passive heat acclimation strategies can accelerate thermal adaptations in females. The large dual thermal stimulus from 6 days of HWI should be sufficient to initiate heat acclimation adaptations in these populations as they typically have smaller body masses than adult males and consequently gain heat more quickly (61). Because of the smaller body masses these future investigations might require shorter maximum HWI durations to cause the beneficial thermal adaptations.

### Perspectives and Significance

Our findings show that medium-term post-exercise HWI confers more complete heat acclimation than conventional exercise heat acclimation, without increasing the risk of overreaching. Compared with conventional exercise heat acclimation, post-exercise HWI caused a greater reduction in resting whole body temperature (core and periphery), which highlights the importance of a large dual (endogenous and exogenous) thermal stimulus for optimizing adaptation to the heat. The consistently reported large reduction in resting core temperature after HWI is most likely explained by the establishment of a new lower thermal balance point and not initiated by thyroid hormone alterations, changes in heat production, or heat loss mechanisms. In addition to lowering resting whole-body temperature, post-exercise HWI also caused more pronounced beneficial exercising thermal and perceptual adaptations than conventional exercise heat acclimation. Future research should assess whether the reduction in thermal strain after post-exercise HWI translates to “real-world” performance improvements and reduces the incidence of exertional heat illness.

## GRANTS

The work was funded by the Chief Scientific Advisers research program, Ministry of Defence UK (to N. P. Walsh and J. A. Mee), through the Defence Science and Technology Laboratory (DSTLX-1000111028).

## DISCLOSURES

No conflicts of interest, financial or otherwise, are declared by the authors.

## AUTHOR CONTRIBUTIONS

R.D.M., M.J.Z., J.A.M., N.P.W., and S.J.O. conceived and designed research; R.D.M., M.J.Z., J.A.M., and N.P.W. performed experiments; R.D.M. and S.J.O. analyzed data; R.D.M., M.J.Z., J.A.M., N.P.W., and S.J.O. interpreted results of experiments; R.D.M. and S.J.O. prepared figures; R.D.M., M.J.Z., J.A.M., N.P.W., and S.J.O. drafted manuscript; R.D.M., M.J.Z., J.A.M., N.P.W., and S.J.O. edited and revised manuscript; R.D.M., M.J.Z., J.A.M., N.P.W., and S.J.O. approved final version of manuscript. 
